# Amniotic Membrane and Mesenchymal Stem Cell Coalescence for Islet Transplantation in Experimental Diabetes in Rats

**DOI:** 10.1155/term/2645595

**Published:** 2025-12-22

**Authors:** Meral Tiryaki, Nurgul Atmaca, Ferda Pinarli, Gulbahar Boyuk Ozcan, Mehmet Sedat Feyat, Sercan Mercan, Aynur Albayrak, Hasan Tarik Atmaca

**Affiliations:** ^1^ National Microbiology Reference Laboratory, Public Health Agency of Türkiye, Ankara, Türkiye; ^2^ Department of Physiology, Faculty of Veterinary Medicine, Balikesir University, Balikesir, Türkiye, balikesir.edu.tr; ^3^ Viagen Laboratory and Health Services, Ankara, Türkiye; ^4^ Department of Physiology, Faculty of Medicine, Ankara Medipol University, Ankara, Türkiye; ^5^ Institute of Science Biotechnology, Middle East Technical University, Ankara, Türkiye, metu.edu.tr; ^6^ Department of Gastroenterology, Gastrointestinal Oncology and Endocrinology, University Medical Center Göttingen, Gottingen, Niedersachsen, Germany, umg.eu; ^7^ Department of Pathology, Ministry of Health Bilkent City Hospital, Ankara, Türkiye; ^8^ Department of Pathology, Faculty of Veterinary Medicine, Balikesir University, Balikesir, Türkiye, balikesir.edu.tr

**Keywords:** amnion membrane, diabetes mellitus, islet cells, mesenchymal stem cell, rat

## Abstract

**Aims:**

The aim of this study is to investigate the effects of islet cells and mesenchymal stem cells transferred together in the amniotic membrane (AM) in order to preserve the viability and functionality of islet cells on the success of islet transplantation in diabetes mellitus–induced rats.

**Methods:**

A total of 80 male Wistar albino rats, aged 3.5–4 months, were included in this study. While 40 Wistar Albino rats were used for the process of islet cell isolation, 40 Wistar Albino rats were used to establish experimental groups. These rats were assigned to five experimental groups including eight rats in each. These groups were AM, amniotic membrane + mesenchymal stem cell (AM + MSC), amniotic membrane + islet cell (AM + IC), amniotic membrane + islet cell + mesenchymal stem cell (AM + IC + MSC), and sham groups. The study was concluded for 28 days.

**Results:**

Although there was no significant difference between AM + IC and AM + IC + MSC groups in terms of mean blood glucose levels, both groups had statistically different values compared to the sham group. A significant difference was observed between the AM + IC and AM + IC + MSC groups in the c‐peptide levels before and after transplantation. Immunohistochemical staining illustrated the presence of insulin‐positive cells in both AM + IC and AM + IC + MSC groups. Moreover, BrDU (+) cells were determined in AM + IC and AM + IC + MSC groups using BrDU staining.

**Conclusion:**

The study results indicated that transplanting islet cells into the omentum by being packaged in AM preserved their viability and function, leading to significant effects on blood glucose and c‐peptide levels.

## 1. Introduction

Type 1 diabetes mellitus is characterized by the autoimmune destruction of insulin‐secreting beta cells in the pancreas, and blood glucose levels of patients with diabetes mellitus are managed through exogenous insulin therapy to maintain normoglycemia [1, 2]. In recent years, allogeneic islet cell transplantation has emerged as an alternative treatment modality [3]. Following islet cell transplantation, the cells are susceptible to damage due to hypoxia, ischemia, and inflammatory responses mediated by immune cells [4, 5].

Numerous studies have reported that the anti‐inflammatory, immunosuppressive, and regeneration‐stimulating effects of mesenchymal stem cells (MSCs) increase the success rate of islet transplantation [6, 7]. Moreover, various studies conducted on several animals with type 1 diabetes mellitus have reported that MSCs reduce inflammation in the host [8, 9] and increase revascularization of the graft [10, 11]. Furthermore, Arzouni et al. [12] showed that culturing islet cells with adipose tissue‐derived MSCs in mice and humans for 48–72 h before transplantation improved the insulin secretion function of the graft. Another study reported that the use of autologous bone marrow–derived MSCs in islet transplantation positively affected the success of transplantation [13].

On the other hand, encapsulation approaches have gained momentum in recent years in order to protect islet cells or MSCs from the attack of the host immune system after transplantation. In the encapsulation process, direct contact of the transplanted cells with the immune system cells is prevented, and selectively permeable membranes are used that allow the transplanted cells to receive the nutrients and gases they need by passive diffusion [14]. Although previous studies reported that islet cells encapsulated with purified alginate [15], hydrogel nanofilm [16], and nanofibrous device [17] could improve the functionality of the graft, the survival rate of islet grafts is still low due to the poor blood supply in the encapsulated islets after transplantation and immune rejection caused by the encapsulated abiotic material. To overcome this shortcoming, biological materials with low immunogenicity are being considered by many groups.

The amniotic membrane (AM), a tissue discarded after cesarean section, is widely used for therapeutic purposes because it is rich in biomolecules that support cell proliferation, especially growth factors [18] and has a low immunogenic structure [19]. Being called as the “true embryonic membrane”, AM consists of epithelium, basement membrane, and dense stroma. It has attracted great attention in tissue engineering and regenerative medicine studies in recent years due to its biocompatible, flexible, antibacterial, anti‐inflammatory, antifibrotic, immunomodulatory, and angiogenic properties. In addition to stem cells, growth factors, and bioactive molecules, the extracellular matrix is of great importance in therapeutic applications [20–22].

Studies investigating different methods to reduce complications have reported that the co‐transplantation of islet cells and MSCs significantly contributes to the preservation of cell mass and maintenance of function [23–25]. Another study has shown that the co‐transplantation of MSCs and islet cells reduces the apoptosis of beta cells and the secretion of proinflammatory cytokines, promotes the proliferation of beta cells, inhibits the activation of antigen‐presenting cells and T cells, and increases the number of CD25+, Foxp3+, and regulatory T cells [26]. In addition, the related studies aiming to increase the success rate of islet cell transplantation have shown that various applications using MSCs sources such as islet cells and human AM MSCs [27] and human AM MSCs/umbilical cord MSCs increase the efficacy of transplantation [28].

One of the most important problems encountered in islet cell transplantation is the loss of vitality and function of islet cells after transplantation. In this study, AM was evaluated as a biological sheath that would protect islet cells from external factors. The present study aimed to examine how islet cell–derived MSCs placed inside this sheath would contribute to the maintenance of vitality and function of islet cells through their paracrine effects. The AM‐MSC‐islet cell structure was transplanted in the form of a sac to the omentum, a region with strong vascularization and nutrition. With this approach, it was aimed to increase the success of transplantation by preserving the vitality and function of islet cells after transplantation.

## 2. Methods

Eighty male Wistar Albino rats (3.5–4 months old) were used in this study after the approval of the animal ethics committee of Diskapi Yıldırım Beyazit Training and Research Hospital was obtained (approval number: 2015/28). Forty of these rats were used for the process of islet cell isolation and the production of MSCs from isolated islets. The remaining 40 rats were used as experimental groups for islet cell transplantation. AM samples were obtained with the permission of the ethics committee of Ondokuz Mayis University Faculty of Medicine Ethics Committee (approval number: 2007/133). AM samples were donated by volunteer expectants and were being stored at −80°C in 50% glycerol (Riedel‐de Haen, Germany) and 50% DMEM (Lonza, Belgium) until the day of the experiment.

### 2.1. Preparation of Islet Cells

For the isolation of pancreatic islets, the rats were anesthetized with xylazine (10 mg/kg) and ketamine hydrochloride (60 mg/kg) and operated on abdominally, and ductus was relieved by applying pressure on the liver. Collagenase type V (7 mL, Sigma, USA) was perfused into the pancreas using a 22 G catheter. The pancreas was removed and immediately placed on ice in a 50‐mL Falcon tube. The material was then incubated at 37°C for 18 min in a water bath, and 25 mL of cold HBSS (+) (10% FBS, 1% L‐glutamine, 1% penicillin–streptomycin–amphotericin B, and 88% HBSS) (Lonza, Belgium) was applied to it and shaken vigorously. The supernatant was removed after centrifugation for 3 min at 4°C. The samples were centrifuged twice. Then, 50 mL of cold HBSS (+) (Lonza, Belgium) was added to the pellet and shaken vigorously. The pancreatic tissue was filtered using a cell strainer (425 μm). The supernatant was removed after 3 min of centrifugation at 4°C, 5 mL of Biochrom 1100 (Biochrom, Germany) was added, and the tissue was homogenized. After homogenization, 10 mL of Biochrom 1077 (Biochrom, Germany) and 10 mL of RPMI 1640 (Lonza, Belgium) were added slowly. The mixture was centrifuged for 20 min at 4°C. The layer consisting of islet cells between the 1077 and RPMI layers was removed carefully into RPMI 1640 (+) (10% FBS, 1% L‐glutamine, 1% penicillin–streptomycin–Amphotericin B, and 88% RPMI 1640) (Lonza, Belgium). The cells were then centrifuged twice for 3 min at 4°C. The cells were counted using the hand‐picking method.

In this study, rats were anesthetized via an intraperitoneal injection of ketamine hydrochloride (60 mg/kg) and xylazine (10 mg/kg). The abdominal area was shaved and disinfected with an antiseptic solution. Following a midline incision, the liver was gently pressed to expose the common bile duct. The pancreas was perfused through a 22G catheter with 7 mL of collagenase type 5 solution (Sigma, USA). The pancreas was then excised and immediately placed on ice in a 50‐mL Falcon tube. Tissue samples were incubated in a water bath at 37°C for 18 min. Afterward, 25 mL of cold HBSS (+) solution (containing 10% FBS, 1% L‐glutamine, 1% penicillin‐streptomycin‐amphotericin B, and 88% HBSS; Lonza, Belgium) was added and the mixture was vigorously shaken. The suspension was centrifuged at 4°C for 3 min. The pellet was resuspended in 50 mL of cold HBSS (+) and mixed thoroughly. The pancreatic tissue was then filtered using a 425‐μm mesh cell strainer. Following another centrifugation at 4°C for 3 min, the supernatant was discarded. The resulting pellet was homogenized with 5 mL of Biochrom 1100 (Biochrom, Germany). Then, 10 mL of Biochrom 1077 (Biochrom, Germany) and 10 mL of RPMI‐1640 (Lonza, Belgium) were slowly layered. The mixture was centrifuged at 4°C for 20 min. The interphase, containing the islet cells, was carefully collected and transferred into RPMI‐1640 (+) medium (containing 10% FBS, 1% L‐glutamine, 1% penicillin‐streptomycin‐amphotericin B, and 88% RPMI‐1640; Lonza, Belgium). The cells were washed twice by centrifugation at 4°C for 3 min. After they were counted under a microscope using the hand‐picking method, they were cultured.

### 2.2. Viability Assessment for Pancreatic Islet Cells

Islet specimens (90 μL) were placed in a Petri dish, and PBS (Lonza, Belgium) was added (910 μL). 20 μL of FDA (Fluorescein Diacetate) stock solution and 20 μL of propidium iodide (PI) stock solution were added to the sequel and stored in the dark for 5 min. The viability test was run using a fluorescent microscope with 40× magnification and analyzed using MATLAB.

### 2.3. Culturing the Isolated Pancreatic Islet Cells

#### 2.3.1. Derivation of MSCs From Pancreatic Islet Cells

The cells were counted using the hand‐picking method, and 2000 islet cells were seeded into T75 flasks in RPMI (+) (Lonza, Belgium) and incubated at 37°C.

Four hundred islets were counted with hand‐picking method, and 200 islets for each flask were inoculated into T75 flasks in MSC medium containing 20% fetal bovine serum (Lonza, Belgium), 2% L‐glutamine (Lonza, Belgium), 1% penicillin‐streptomycin‐amphotericin B (Lonza, Belgium), and 77% DMEM (Lonza, Belgium). The medium was changed every 2–3 days, and cell growth was observed under an inverted microscope (Leica Inverted Microscope, Germany). Cell passaging was performed after 10 days. MSCs obtained after the second passage were differentiated. MSC characterization was performed by determining MSC‐specific surface markers through flow cytometry.

After islet cell isolation, 200 islet cells were cultured in MKH medium in each of T25 flasks. The culture medium was refreshed every 2–3 days, and cell growth was monitored using a Leica inverted microscope. At the end of the second passage, the obtained MSCs were subjected to differentiation protocols. Flow cytometric analysis was performed to identify MSC surface markers. Remaining cells were preserved in cryopreservation solution and stored at −86°C. The MSCs used in this study were derived from the same pancreatic islet cell isolates obtained from the aforementioned rats. Following isolation, the islet cells were cultured in MSC‐specific medium, and MSCs were obtained after the second passage and characterized by flow cytometry.

### 2.4. Differentiation of MSCs

#### 2.4.1. Characterization of Islet Cell–Derived MSCs

To differentiate MSCs into adipocytes, osteocytes, and chondrocytes, specific basal media (Gibco, USA) and supplements were used for each cell type. The medium of the cells was changed every 3 days after the second passage. At the end of the third week, oil red staining (Diagnostıc BioSystem, USA) for adipocyte differentiation, von Kossa staining (Diagnostıc BioSystem, USA) for osteocyte differentiation, and Alcian blue staining (Diagnostıc BioSystem, USA) for chondrocytes were performed using an inverted microscope to detect differentiated cells (Figure 1).

Figure 1Differentiation of MSCs; (a, b) Obtaining MSCs from Islet Cells at 3 days (magnification: 4×), (c) MSCs at Passage 2 (magnification:4×), (d) differentiation of MSCs into adipocytes (oil red staining, Magnification:40×), (e) differentiation of MSCs into osteocyte (von Kossa staining, magnification: 10×), (f) differentiation of MSCs into chondrocyte (Alcian blue staining, magnification: 10×). Scale bars are present in each panel.

**Figure:**
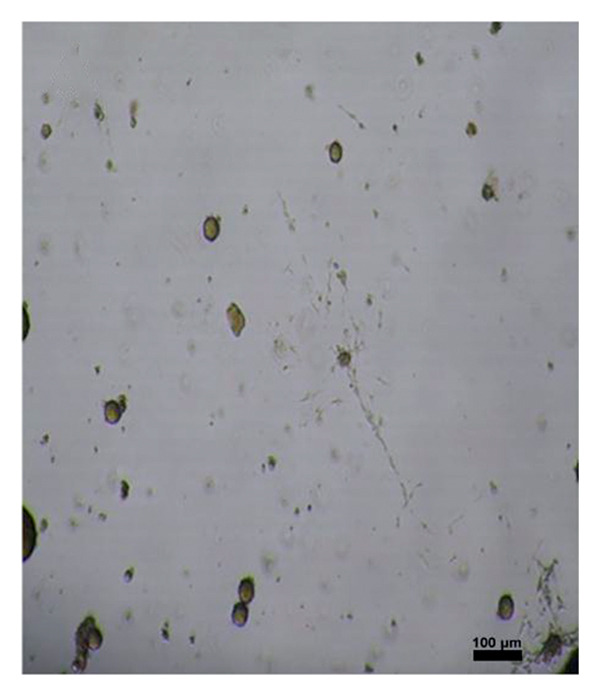
(a)

**Figure:**
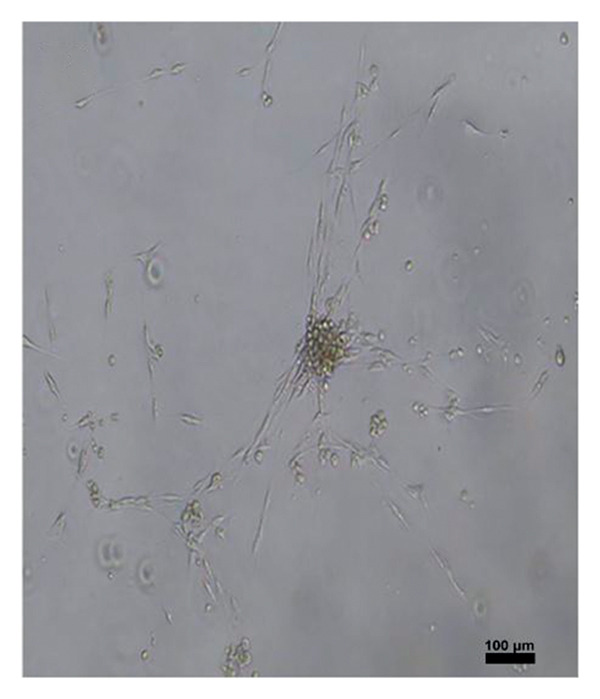
(b)

**Figure:**
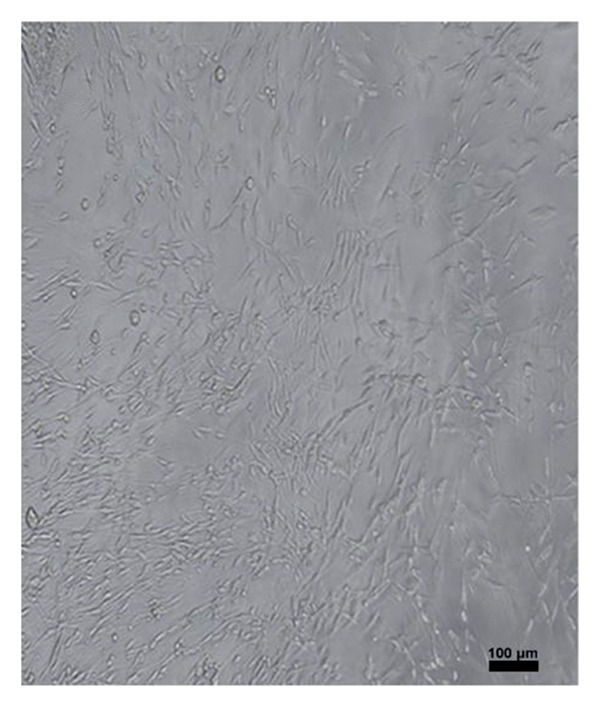
(c)

**Figure:**
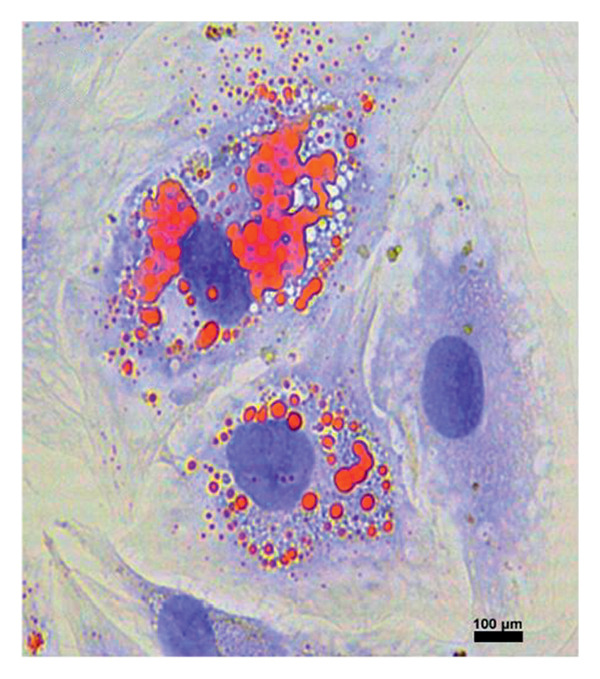
(d)

**Figure:**
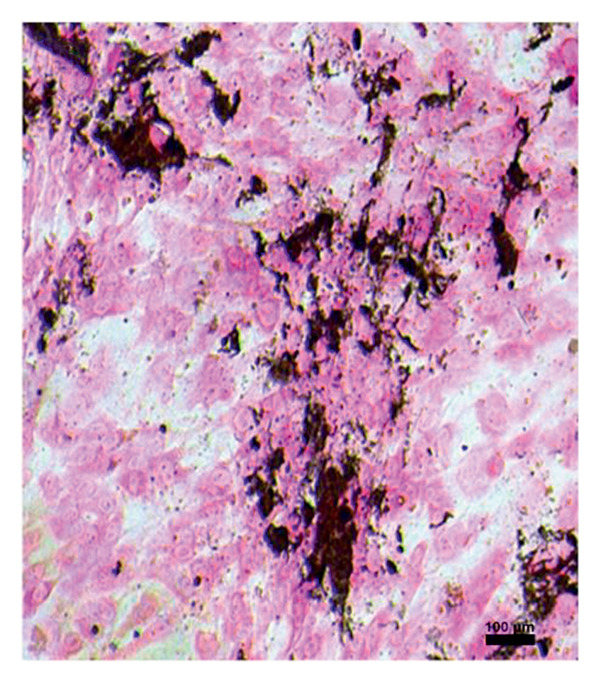
(e)

**Figure:**
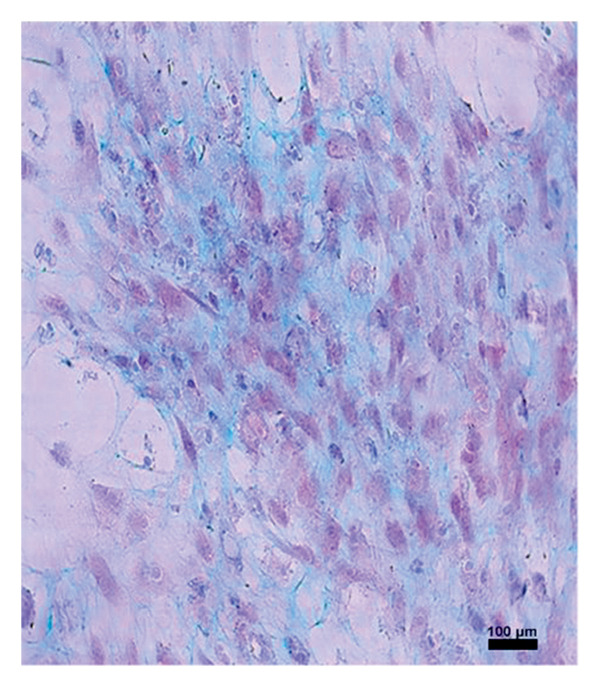
(f)

At the end of the second passage, cells were suspended and loaded into a flow cytometer (FACS‐ARIAIII, BD, USA) in three tubes containing 15 × 10^4^ cells. The cell suspension was centrifuged in PBS for 5 min at 1200 rpm and incubated in the dark for 45 min after the addition of antibodies against CD45R, CD11b/c, CD90, and CD44 (BD, USA). After incubation, each tube was read on a flow cytometry device.

The results were expressed as follows: CD45R (5.7% (−); CD11b/c (4.5% (−), CD90 (90.8% (+); and CD44 (85% (+) (Figure 2).

Figure 2Characterization of the MSCs with surface markers using flow cytometry: (a) CD45R PE‐A isotypic control, (b) CD 11b/c FITC‐A isotypic control, (c) CD90 PE‐A (+), CD44 FITC‐A (+), (d) CD11b/c FITC‐A (−), CD45 PE‐A (−).

**Figure:**
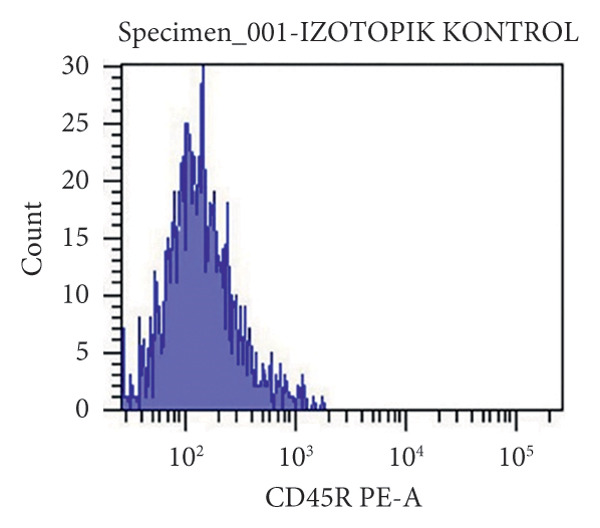
(a)

**Figure:**
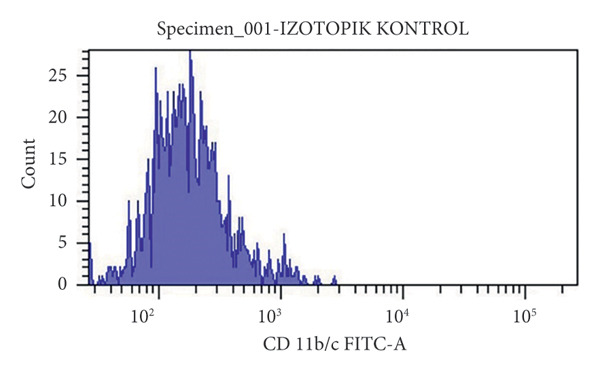
(b)

**Figure:**
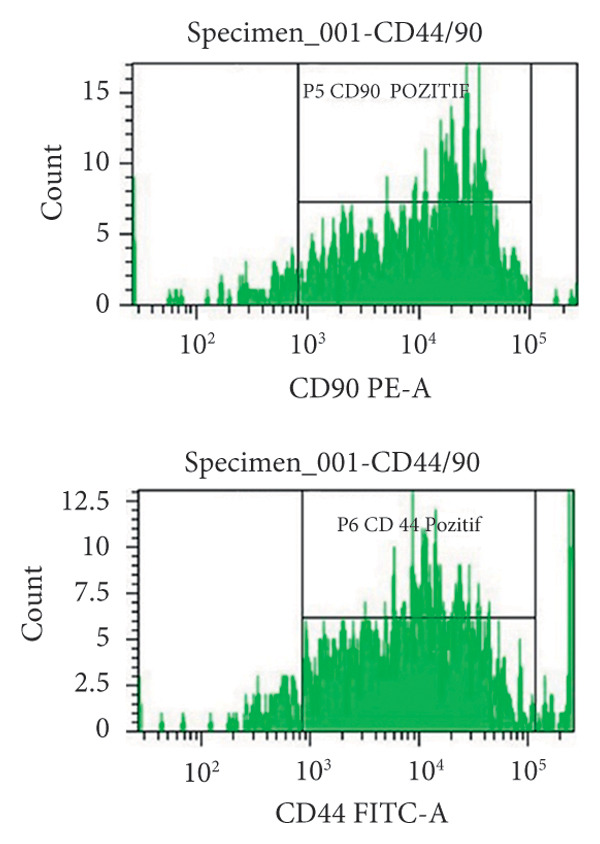
(c)

**Figure:**
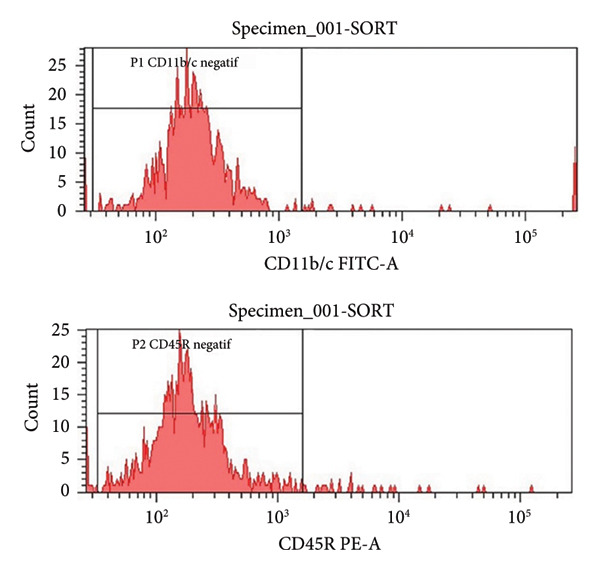
(d)

#### 2.4.2. Islet Cell Culture

Islets counted using the hand‐picking method were incubated in islet cell culture medium at 37°C in a 5% CO_2_ atmosphere, with 2000 islet cells inoculated per T75 culture flask, 24 h prior to transplantation.

### 2.5. Preparation of Amnion Membrane

For all groups, AM material stored at −80°C was first solubilized in a water bath at 37°C for 1–2 min. DMEM (Lonza, Belgium) was added to AM and washed twice. After brief washing, the chorionic layer on AM was separated by mechanical methods [29]. De‐epithelization of the AM was performed as follows: AM was soaked in trypsin‐EDTA‐C (Lonza, Belgium) at 37°C for 30 min; trypsin‐EDTA was removed using DMEM consecutively, and AM samples were transferred to 3.5% paraformaldehyde (PFA) solution. Afterward, the tissues fixed with PFA were placed in sucrose solution and tissue tracking procedures were performed. De‐epithelialized AM samples (Figure 3) were cut into 1‐cm‐diameter pieces. These pieces were stored in a storage solution containing 50% glycerol and 50% L‐DMEM in a deep freezer at −86°C until in vivo studies were conducted [30].

Figure 3Nuclear labeling with Hoechst33258 (blue color) of fresh (a) and de‐epithelialized (b) amniotic membrane. Zeiss LSM880 confocal microscope (magnification: 40×). Scale bars are present in each panel.

**Figure:**
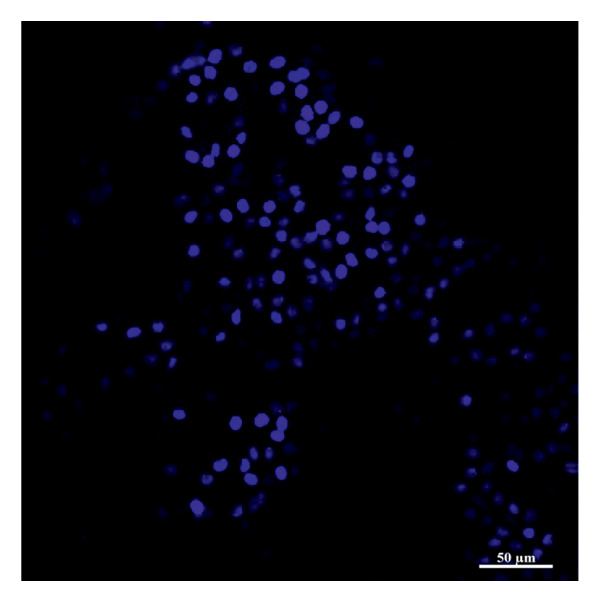
(a)

**Figure:**
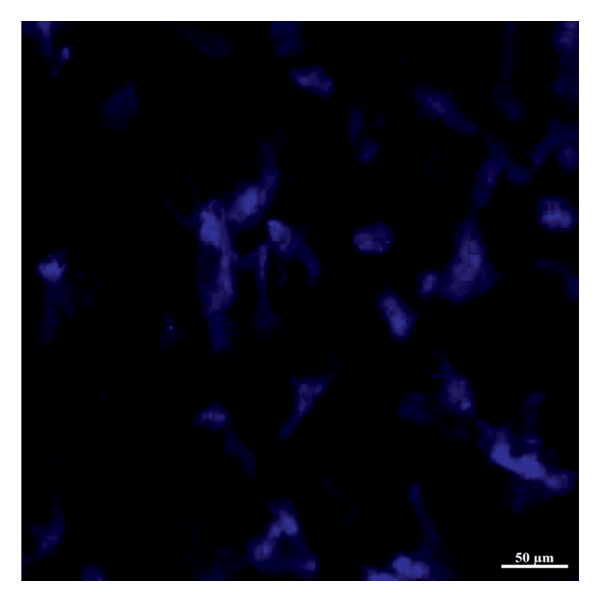
(b)

### 2.6. Induction of Diabetes Mellitus by Streptozotocin

After 12 h of fasting, streptozotocin (45 mg/kg) was intraperitoneally injected into male Wistar Albino rats. Rats having blood glucose levels ≥ 250 mg/dL 3 days after streptozotocin administration were considered as diabetic. Blood glucose levels were measured twice a week for 15 days following the application, and those having a blood glucose of ≥ 250 mg/dL were considered as diabetic and included in the transplantation procedures.

### 2.7. Experimental Groups

#### 2.7.1. Experimental Groups

There were five groups [amnion membrane (AM), amnion membrane + mesenchymal stem cell (AM + MSC), amnion membrane + islet cell (AM + IC), amnion membrane + islet cell + mesenchymal stem cell (AM + IC + MSC), and sham] which had 8 animals in each. All those diabetic rats were anesthetized with xylazine (10 mg/kg) and ketamine hydrochloride (60 mg/kg), and omentum was designated after abdominal opening. Omentum membrane was spread onto a sterile wet sponge for transplantation or sham operation. The surgical operations and specifications of the groups are as follows:

AM: The AM material removed at −86° was kept in a 37° water bath for 2 min, and the transformation of the solution from solid to liquid was observed. AM material was placed in another Falcon tube and sequential washing procedures were performed. A 1‐cm‐diameter AM material was incubated in MKH medium. AM material, which was incubated for 24 h, was engrafted onto the omentum membrane, and omentum was sutured as a sac. Subcutaneous and cutaneous tissues of the rats were sutured.

AM + MSC: The AM material removed at −86° was kept in a 37°C water bath for 2 min, and the transformation of the solution from solid to liquid was observed. AM material was placed in another Falcon tube, and sequential washing procedures were performed. Cryopreserved islet‐derived MSCs were solubilized by using a water bath of 37°C. The cells were centrifuged for 10 min at 1,200 rpm, the supernatant was removed, and DMEM was added again for washing the cells. This step was repeated twice. The cells were counted and viability test was run with Countess (Invitrogen, Australia). A 10μM BrDU (BD, USA) solution was prepared in 1 mL. A 10 μL BrDU solution was added onto the cells (uttermost 2 × 10^6^ cells/cc). This suspension was incubated for 2 h, at 37°C, 5% CO_2_. The 5 × 10^4^ islet‐derived MSCs were pipetted onto a 1‐cm‐diameter AM Petri dish. MSC medium was added to the Petri dish with 5–10 min interval. The prepared cells were incubated in 5% CO_2_ at 37°C for 24 h before transplantation. The prepared AM + MSC material was engrafted onto omentum membrane of the rats in the way that the cells placed onto the upperpart and omentum was sutured as a sac. Subcutaneous and cutaneous tissues of the rats were sutured.

AM + IC: After islet cell isolation cells were counted with hand‐picking method, and 2000 islet cells per flask were seeded and incubated in 5% CO_2_ at 37°C. Cultured islet cells were collected and washed twice with PBS and centrifuged at 1300 rpm for 5 min. AM samples which were incubated for 24 h were prepared as mentioned before. The prepared AM material was engrafted onto omentum membrane, and islet cells were dropped onto the material with a Pasteur pipette. Omentum was sutured as a sac. Subcutaneous and cutaneous tissues of the rats were sutured.

AM + IC + MSC: The AM material removed at −86° was kept in a water bath of 37°C for 2 min, and the transformation of the solution from solid to liquid was observed. AM material was placed in another Falcon tube, and sequential washing procedures were performed. Cryopreserved islet‐derived MSCs were solubilized by using a water bath of 37°C. The cells were centrifuged for 10 min at 1200 rpm, the supernatant was removed, and DMEM was added again for washing the cells. This step was repeated twice. The cells were counted, and viability test was run with Countess (Invitrogen, Australia). A 10μM BrDU (BD, USA) solution was prepared in 1 mL. A 10 μL BrDU solution was added onto the cells (uttermost 2 × 10^6^ cells/cc). This suspension was incubated at 37°C in 5% CO_2_ for 2 h. The 5 × 10^4^ islet‐derived MSCs were pipetted onto a 1‐cm‐diameter AM Petri dish. MSC medium was added to the Petri dish with 5–10 min interval. The prepared cells were incubated at 37°C in 5% CO_2_ for 24 h before transplantation. Then, the islet cell isolation cells were counted with hand‐picking method and 2,000 islet cells per flask were inoculated and incubated at 37°C in 5% CO_2_. Cultured islet cells were collected and washed twice with PBS, centrifuged at 1,300 rpm for 5 min, and incubated for 24 h. The prepared AM and MSC were engrafted onto omentum membrane, and islet cells were dropped onto the material with a Pasteur pipette. Omentum was sutured as a sac. Subcutaneous and cutaneous tissues of the rats were sutured.

Sham: Diabetes‐induced rats were anesthetized with xylazine (10 mg/kg) and ketamine hydrochloride (60 mg/kg), and omentum was designated after abdominal opening for sham operation. Omentum was sutured as a sac. Subcutaneous and cutaneous tissues of the rats were sutured.

### 2.8. Measurement of Blood Glucose Level

Blood glucose levels were measured using a glucometer as follows: the first 3 days after transplantation and at certain times (09:00–10:00), twice a week after the first 3 days. The experiments were terminated 4 weeks after transplantation (GlucoDr, Korea).

### 2.9. Intraperitoneal Glucose Tolerance Test (IPGTT)

The IPGTT was run on the 26th day after transplantation. The rats were weighed a day before the test and unfed for 12 h. The pre‐prandial blood glucose levels of the rats were measured. Then, glucose (i.p., 2 g/kg) was injected into the rats, and their blood glucose levels were measured at the 5th, 10th, 15th, 30th, 60th, 90th, and 120th min after glucose injection.

### 2.10. Microscopic Examination and Immunohistochemistry

All of the tissue samples were blocked in paraffin after 24 h of fixation in 10% buffered neutral formalin solution and then cleared in xylene and paraffin‐embedded tissue sections. After hematoxylin and eosin (H&E) staining, immunohistochemical analyses were done with BrDU (BD, USA) and insulin antibodies using 5‐μm sections of the tissues. All the procedures were done at room temperature, and the results were evaluated using a light microscope.

### 2.11. C‐Peptide Analysis

First, the serum proportion of 1 mL blood was separated for the evaluation of c‐peptide level. The sandwich ELISA test was done on the same day using ELISA kits (Millipore, USA). Enzyme activities were measured spectrophotometrically.

### 2.12. Statistical Analysis

The SAS (SAS version 8.02, SAS Institute, Cary, NC, USA) software was used for statistical analysis. Repetitive measurements were analyzed using a general linear model (GLM). The Tukey test was used to determine the intergroup and intragroup differences, and the value of *p* < 0.05 was considered statistically significant. All results were given as mean ± standard error of mean (SEM).

## 3. Results

### 3.1. Viability of Islet Cells

According to the viability analyses conducted based on the MATLAB software, the average viability rate of the AM + IC group was 96.74%, whereas the average viability rate of the AM + IC + MSC group was 95.87%.

### 3.2. The Mean Blood Glucose Levels of the Groups

Figure 4 shows mean blood glucose values measured on the days before and after transplantation. There were no significant differences between the groups for the mean pre‐transplant blood glucose values. In comparison to sham, it was observed that there were no statistical differences for the average blood glucose values of AM. It was found that a significant difference between AM + MSC and sham groups was observed only on day 2, while no significant differences were detected on the other days. On the other hand, all post‐transplant measures revealed that the average blood glucose values of the AM + IC + MSC and sham groups were statistically different, and the average blood glucose values of the AM + IC and sham groups were statistically different except on days 9 and 16.

Figure 4Mean blood glucose levels on the pre‐ and post‐transplantation days. Blood glucose levels were measured 3 days after transplantation and twice a week thereafter, with experiments ending 4 weeks post‐transplantation. (a) Blood glucose levels in pre and post days of transplantation. (b) Blood glucose levels over time. All data presented as mean ± SEM. ^∗^ *p* < 0.05; *n* = 8 per group. The following statistical tests were done: general linear model (GLM) and the Tukey test.

**Figure:**
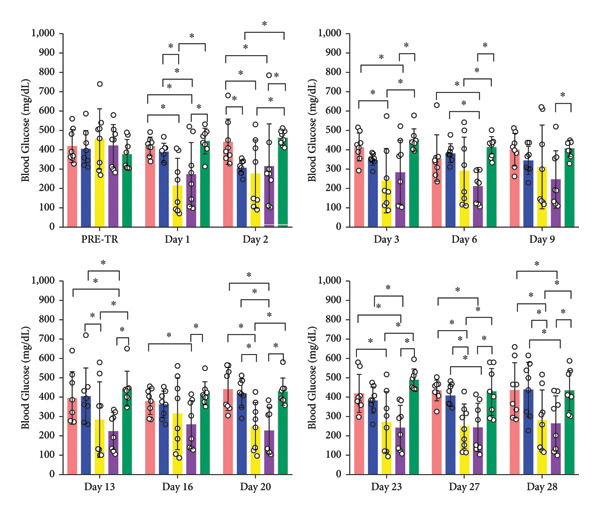
(a)

**Figure:**
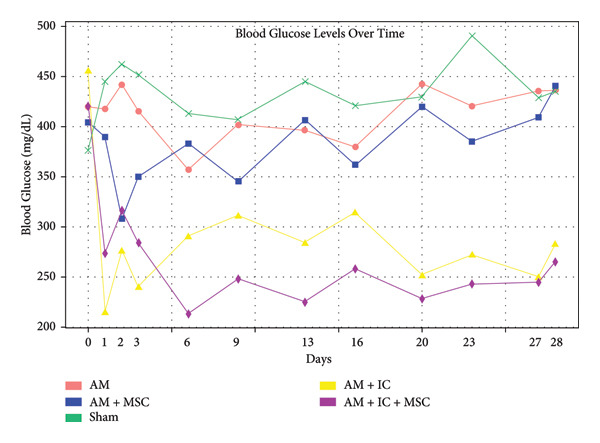
(b)

### 3.3. C‐Peptide Test

**Figure 5:**
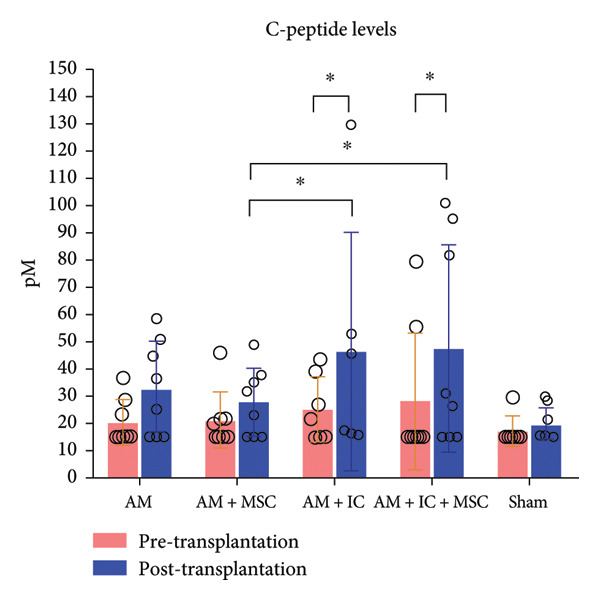
Pre‐ and post‐transplantation mean C‐peptide levels of the groups. Serum proportion of 1 mL blood was separated for c‐peptide level evaluation, followed by sandwich ELISA test using ELISA kits and spectrophotometric measurement of enzyme activities. All data presented as mean ± SEM. ^∗^ *p* < 0.05; *n* = 8 per group. The following statistical tests were done: general linear model (GLM) and the Tukey test.

The average C‐peptide values pre‐ and post‐transplant are presented in Figure 5. The pretransplant values of the groups were not significantly different from one another. AM, AM + IC, and AM + IC + MSC groups exhibited statistically significantly higher post‐transplant C‐peptide levels than the sham group. Values of C‐peptide were found to be significantly different between the AM + IC and AM + IC + MSC groups both pre‐ and post‐transplant.

### 3.4. IPGTT

IPGTT blood glucose averages of study groups are provided in Figure 6. IPGTT blood glucose averages of AM + MSC, AM + IC, and AM + IC + MSC at the 0th, 5th, 10th, 15th, 30th, 60th, 90th, and 120th min were statistically different compared to the sham group. In addition, mean IPGTT blood glucose levels of the AM group were different from those of the sham group only at the 5th, 10th, and 15th min.

Figure 6IPGTT blood glucose levels. The IPGTT was conducted on the 26th day after transplantation, with rats weighed and unfed for 12 h before glucose injection. Blood glucose levels were measured after 5th, 10th, 15th, 30th, 60th, 90th, and 120th minutes. (a) IPGTT blood glucose levels in different times. (b) Mean IPGTT blood glucose levels over time. All data presented as mean ± SEM. ^∗^ *p* < 0.05; *n* = 8 per group. The following statistical tests were done: general linear model (GLM) and the Tukey test.

**Figure:**
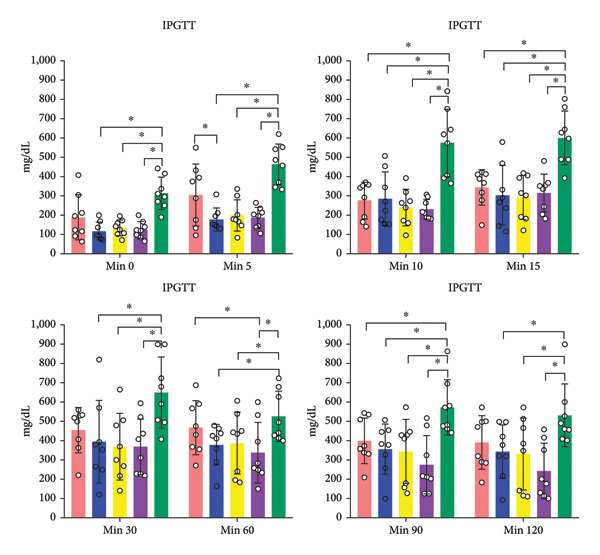
(a)

**Figure:**
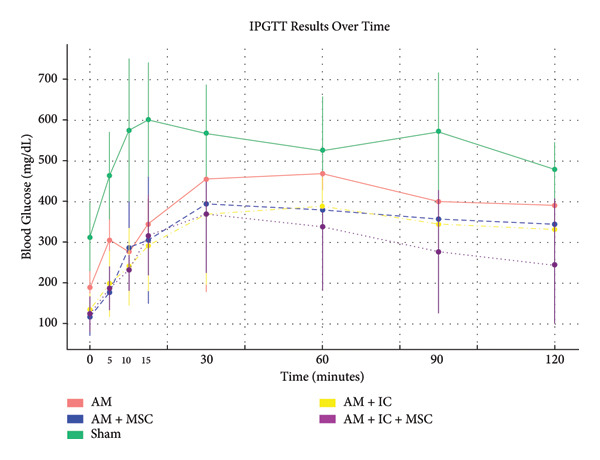
(b)

### 3.5. Immunohistochemical Assay

In the study, the omenta of all groups were subjected to HE staining. Omentum tissue and AM were observed in AM group. In the AM + MSC group, omentum and AM were observed, whereas MSC was determined by BrDU staining. In the AM + IC group, islet cells, omentum, and AM were detected by HE staining, while cells that react with insulin were detected by an immunohistochemical study conducted with anti‐insulin antibody. In the AM + IC + MSC group, islet cells, omentum, and AM were observed by HE staining, whereas immunohistochemically, MSC was observed with BrDU staining and islet cells were observed by the study conducted with insulin antibody. After BrDU staining, MSCs clustered around islet cells; moreover, MSCs and islet cells created a complete unit. In the sham group, omentum was detected by HE staining (Figure 7).

Figure 7(a) AM + IC HE staining. Arrows indicate the islet cells (magnification: 40×). (b) AM + IC insulin staining. Arrows indicate the secreted insulin (magnification: 20×). (c) AM + IC + MSC HE staining. Arrows indicate the islet cells. (magnification: 10×). (d) AM + IC + MSC insulin staining. Arrows indicate the secreted insulin (magnification: 10×). (e) AM + MSC immunohistochemical BrDU staining. Arrow indicates MSC (BrDU+) (magnification: 40×). (f) AM + IC + MSC immunohistochemical BrDU. Black arrows indicate islet cells, red arrows indicate MSCs (BrDU+) (magnification: 4×). Scale bars are present in each panel.

**Figure:**
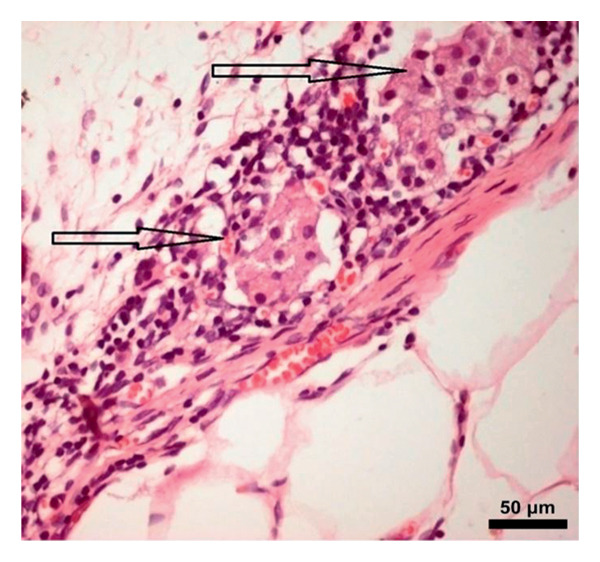
(a)

**Figure:**
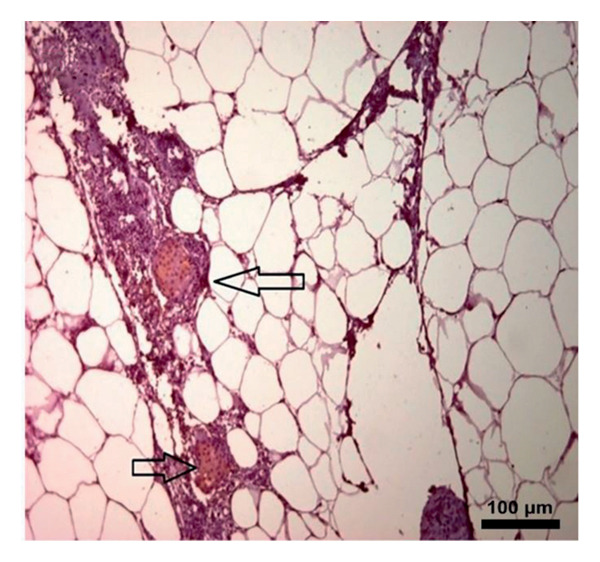
(b)

**Figure:**
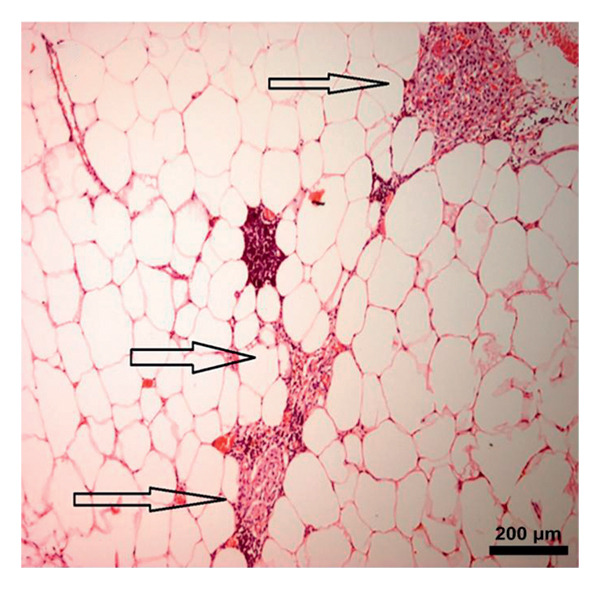
(c)

**Figure:**
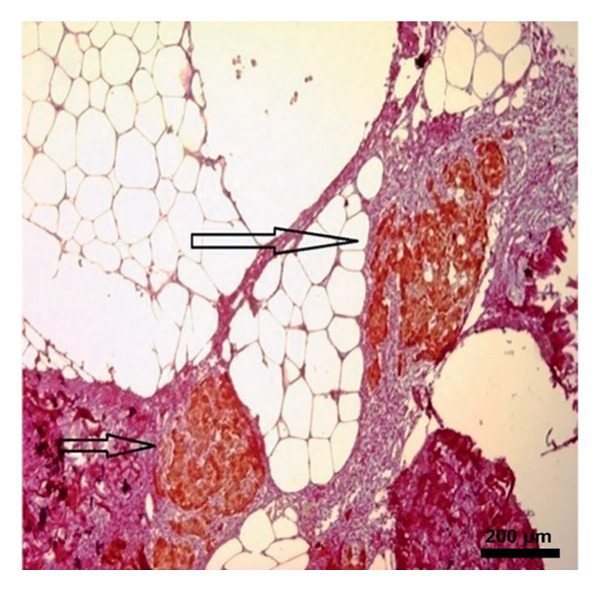
(d)

**Figure:**
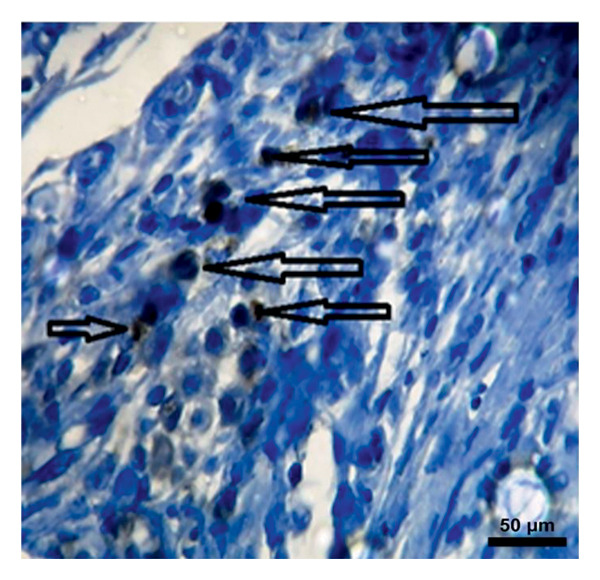
(e)

**Figure:**
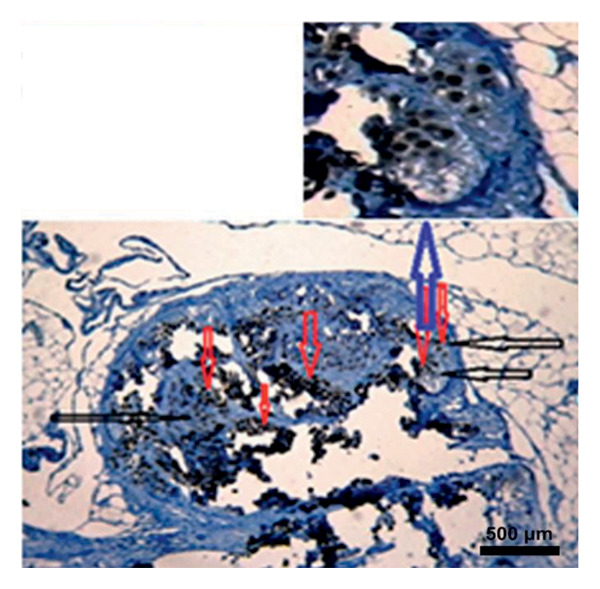
(f)

## 4. Discussion

The replacement of exogenous insulin is a commonly used clinical treatment modality for Type 1 diabetes mellitus, which is characterized by the degeneration of beta cells [31]. However, since long‐term insulin use causes some complications, alternative treatments that can be used instead of insulin have been studied. Transplantation of pancreatic islet cells from cadaveric donors is the most studied treatment method [32]. However, the loss of cells because of oxidative stress associated with insufficient oxygenation, depletion of nutrient supply, inadequate vascularization profile, CD4+ T lymphocytes, CD8+ T lymphocytes, and inflammatory attacks by natural killer cells in the early post‐transplant period of transplanted beta cells [4, 5, 33] has led to the emergence of a new field of research [34]. This field focuses on developing strategies to protect the transplanted beta cells and improve their survival rates. Researchers have investigated various approaches, including encapsulation techniques, immunomodulation, and the use of antioxidants to mitigate oxidative stress. These advancements aim to enhance the efficacy of islet cell transplantation and provide a more sustainable treatment option for individuals with Type 1 diabetes mellitus.

In animal studies conducted so far, different cells such as MSC [35], stellate cells [36], neural crest [37], and human AM MSCs [27, 28], have been transplanted together with islet cells to different body parts, such as tail vein, renal capsule, portal vein, and omentum, with the use of biocompatible, cell mass–supportive tissue scaffolds [38]. Moreover, studies have been conducted on the production of beta cells derived in vitro from embryonic stem cells and induced pluripotent stem cells [39, 40]. These studies have shown promising results in improving survival and function of islet cells after transplantation. However, challenges remain in optimizing the integration of transplanted cells with the host tissue and ensuring long‐term graft survival. Further research is needed to develop standardized protocols for cell preparation, transplantation techniques, and post‐transplant monitoring to enhance the clinical applicability of these approaches.

In this study, AM, which is an immunomodulatory, antimicrobial, bioadaptive, and natural biomaterial [41, 42], was used as a supportive primary transplantation component for islet cells against apoptotic factors that cause islet cell loss in the post‐transplantation period. In the study model, functional islet cells isolated from rats were encapsulated in human AM and transplanted into the omentum of rats with diabetes mellitus. Yang et al. [14] reported that the addition of AM extract, a natural biomaterial, to the culture medium of islet cells before transplantation improved survival and functionality of islet cells after transplantation and reduced islet apoptosis and cell loss. In another study, it was reported that transplantation success in rats with diabetes mellitus increased when human amniotic epithelial cells and islet cells were infused into the AM and transplanted together [43]. Insulin‐producing organoids in the form of islet cells surrounded by AM epithelial cells designed by Lebreton et al. [44] were able to maintain their viability in the hypoxia environment in vitro and continue to function after transplantation into diabetes‐induced mice. In addition, in this study, various analyses such as blood glucose monitoring, c‐peptide test, and IPGTT were performed in order to determine the effect of transplantation on the model and whether this effect was due to the transplanted cell model, which is compatible with similar studies. In addition, the basic activities of the transplanted cells and the transplant model were demonstrated immunohistochemically.

In the present study, the AM samples used for transplantation were used after the decellularization process. Therefore, it is assumed that the support the AM group was thought to provide therapeutic effect was independent of the stem cells in its structure. The omentum was chosen as the site for islet cell transplantation in this study because it provides adequate vascularization and has growth factors [45–47]. Assessments of post‐transplantation blood glucose and c‐peptide levels showed that the transplanted islet cells functioned effectively. Immunohistochemical studies have confirmed this, demonstrating insulin secretion and preservation of three‐dimensional structures. The results of this study suggest that AM provides a protective environment for the transplanted islet cells, potentially increasing their survival and function. This approach may offer a promising alternative to traditional islet cell transplantation methods and may improve outcomes for patients with diabetes mellitus undergoing islet transplantation.

Studies involving the co‐transplantation of islet cells and MSCs have reported that MSCs release a number of growth factors, which increase vascularization [48, 49]. Moreover, MSCs stimulate beta cell proliferation, reduce the apoptosis of the graft, prevent the infiltration of activated immune cells to the graft, and inhibit the release of pro‐inflammatory cytokines [26]. Katuchova et al. [50] demonstrated a blood glucose–lowering therapeutic effect of MSCs in their diabetes model by transplanting MSCs to the different pancreatic microenvironments. Considering the use of MSCs for therapeutic purposes in diabetes models [51, 52], their ability to create paracrine effects [53, 54], and the effect of paracrine effects on healing, it was concluded that MSCs used in this study could increase the success rate of transplantation. These findings suggest that the combination of AM and MSCs could provide a synergistic effect in islet cell transplantation. The protective properties of AM, coupled with the growth factors and immunomodulatory effects of MSCs, may enhance survival and function of islet cells.

In this study, the blood glucose measurements taken after the transplantation revealed that the values of AM + IC + MSC and AM + IC groups were significantly lower than those of the sham group except for the days 9 and 16. In addition, statistical differences were found for the mean IPGTT blood glucose values of the sham group compared to the AM + MSC, AM + IC, and AM + IC + MSC groups. These findings showed that islet cells in the AM + MSC, AM + IC, and AM + IC + MSC groups were sensitive to glucose, and when islet cells were stimulated by glucose, they secreted insulin; therefore, they mediated the regulation of blood glucose. Moreover, it was considered that detection of the insulin (+) islet cells in the AM + IC and AM + IC + MSC groups by immunohistochemical staining was correlated with the presence of islet cells that maintained their viability and functionality. These results suggest that the transplanted islet cells in the AM + IC and AM + IC + MSC groups remained viable and functional, contributing to improved glucose regulation. The presence of insulin‐positive cells in these groups further supported the effectiveness of the transplantation procedure.

The functionality of beta cells cannot be evaluated based on endogenous insulin levels because endogenous insulin displays a pulsatile oscillatory pattern and has a short half‐life. To assess the functionality of beta cells, c‐peptide level, which is an indicator of the production of endogenous insulin and has a longer life span, should be investigated [55]. Neither intragroup nor intergroup differences in c‐peptide levels were detected before and after transplantation among sham, AM, and AM + MSC groups, while it was found that c‐peptide levels detected after transplantation between AM + IC and AM + IC + MSC groups were statistically higher than the levels of the sham group. Moreover, post‐transplant C‐peptide levels of the AM + IC and AM + IC + MSC groups elevated compared to pretransplant C‐peptide levels. These findings suggested that the transplantation of islet cells, either alone or in combination with MSCs, can enhance functionality of beta cells in diabetic recipients. The elevated C‐peptide levels observed in the AM + IC and AM + IC + MSC groups indicate a potential improvement in endogenous insulin production.

## 5. Conclusion

Consequently, to protect the islet cells from immune attack after transplantation, the study was conducted with different transplantation groups based on the idea that MSCs with immunomodulator properties can help islet cells survive and function properly in ways similar to how pancreas‐derived MSCs do this. Because of several parameters, including blood glucose level, blood C‐peptide level, and IPGTT, used to assess the functionality of islet cells alongside the presence of immunohistochemically stained insulin (+) islet cells, it was considered that the AM and MSCs used in the study had positive impacts on the success of transplantation. Moreover, investigating cellular mechanisms through which the biological structure of AM produces this therapeutic effect requires further study as a novel research subject in this field. Further research could explore the specific molecular pathways through which AM and MSCs enhance survival and function of islet cells. Additionally, long‐term studies are needed to evaluate the durability of these protective effects and any potential adverse outcomes. Such studies could pave the way for developing more effective and safer islet cell transplantation protocols for treating diabetes mellitus.

## Disclosure

This study is summarized from Meral Tiryaki’s thesis done in Kirikkale University.

## Conflicts of Interest

The authors declare no conflicts of interest.

## Funding

This study was supported by Kirikkale University Scientific Research Unit with project number 2014/123 and Research Laboratory of Diskapi Yildirim Beyazit Training and Research Hospital.

## Data Availability

The data that support the findings of this study are available from the corresponding author upon reasonable request.
